# The core taxa connectivity in leaf microbiomes in ash trees stands is modulated by host-related and environmental factors

**DOI:** 10.1186/s40793-026-00967-8

**Published:** 2026-10-07

**Authors:** Valentin Burghard, Sonja Wende, Georgia Kahlenberg, Henriette Häuser, Ralf Kätzel, Steffen Kolb, Andreas Ulrich

**Affiliations:** 1 Microbial Biogeochemistry, Leibniz Centre for Agricultural Landscape Research (ZALF), Müncheberg, Germany; 2 Thaer-Institute, Humboldt University of Berlin, Berlin, Germany; 3 Physical Geography/Landscape Ecology and Sustainable Ecosystem Development, Catholic University of Eichstätt-Ingolstadt, Eichstätt, Germany; 4 Department of Forest Ecology and Monitoring (LFE), State Forestry Institute Brandenburg, Eberswalde, Germany

**Keywords:** *Fraxinus excelsior*, Microbiome, Abiotic factors, Ash dieback, Network analysis, Core microbiome

## Abstract

**Background:**

Forests cover large areas of the terrestrial land surface and play a key role in climate regulation and biodiversity conservation. Tree-associated microbial communities are integral components of forest ecosystems and contribute to plant functioning, stress tolerance, and interactions with the environment. However, the factors shaping the phyllosphere microbiome of forest trees across large environmental gradients remain less explored. Here, we investigated the bacterial leaf microbiome of *Fraxinus excelsior* across different climatic regions in Germany to identify its core microbiome and determine the relative importance of environmental and host-related factors shaping the phyllosphere microbiome of forest trees across large environmental gradients assembly. Therefore, 30 ash trees were sampled at each of the 12 sites across Germany, at which we assessed their leaf microbiome, leaf physio-chemical properties and a set of site parameters.

**Results:**

In total, 755 genera were detected. Only three genera *Hymenobacter*, *Massilia* and *Sphingomonas* were predominant across all sites. Geographical position, precipitation, tree age and the leaf and soil chemistry parameters were significant factors that explained 22.1% of variance between microbiome structures between the different sites. Seven genera were identified as the core leave microbiome of *F. excelsior*. The core microbiome assessed for each sampling site was mostly more diverse and differed between sites. Complex co-occurrence networks separated by sites with the identified six hub bacterial taxa proved that they were essential for the local core microbiomes. However, the network complexity differed significantly between the sampling sites.

**Conclusion:**

Our findings clearly indicate the existence of an ash core microbiome on leaves that occurred at all sites but was locally differently interconnected to further microbiome members. The network analyses revealed that less frequent genera play important roles as hub taxa. Furthermore, we were also able to prove the modulating role of biotic and abiotic factors on the ash phyllosphere microbiome. An optimized core microbiome may be considered crucial for improving tolerance against plant diseases, such as ash dieback, in future.

**Supplementary Information:**

The online version contains supplementary material available at https://doi.org/10.1186/s40793-026-00967-8.

## Background

Forests cover approximately 35–40% of Europe’s land surface and play a crucial role in regulating climate and maintaining biodiversity. They are essential for mitigating climate change and are affected by climate change and other negative influences [1]. These pressures affect not only trees themselves but also the diverse microorganisms associated with their tissues and surfaces. Therefore, trees should be considered as holobionts, the unity of the host plant, its organs and microorganisms living within and on them [2]. The plant hosts microorganisms in the phyllosphere, endosphere, and rhizosphere. The microbiome is defined as a specific microbial community within a distinct habitat with unique physiochemical properties [3]. It includes fungi, archaea, bacteria, protists, and algae, which are recruited from soil, air, other plants, and flowers or transferred via seeds [4].

Compared with phyllosphere microbiome of herbaceous plants, the phyllosphere microbiome of trees presents unique, distinguishable characteristics. With their larger surface area, long lifespan, and stratified canopies, trees create diverse habitats with varying microclimatic conditions, leading to greater microbial diversity and specialization compared with those of annual plant species. The microbiomes of trees are shaped by combinations of multiple biotic and abiotic factors, including climate conditions (temperature, humidity and UV radiation), anthropogenic influences (irrigation, pesticides and fertilization), and soil properties (e.g., pH and cation exchange capacity) [4]. Additionally, plant species and plant genotype play key roles in determining microbial community structure [5].

Individual tree-specific factors such as age, canopy structure, and height create gradients of light, temperature, and humidity that further modulate microbial colonization and community composition [6]. Moreover, trees interact with a wider array of environmental factors and organisms, including epiphytic plants, other trees, insects, birds, and the surrounding soil, all of which contribute to the complexity and diversity of their phyllosphere microbiome [7].

Plant–microbiome and microbe–microbe interactions constitute a complex network of the involved partners. The microbiome or some of its specific members may enhance plant growth, health, and stress resistance or mediate induced systemic resistance (ISR) for stronger pathogen defense [8, 9]. Furthermore, microbes also compete for limited resources, occasionally producing secondary metabolites to inhibit others [10]. This competition can benefit plants by suppressing pathogens [11]. Some microbes exploit plant immune responses, such as salicylic acid production, to their advantage [12].

As ash dieback was identified as a major threat to *Fraxinus excelsior*, its microbiome subsequently became the focus of research. Several studies analyzed the structure of bacterial and fungal communities on *F. excelsior* first Scotland and England [13] and in northeastern Germany [14, 15]. These studies concerning the *F. excelsior* microbiome have focused mostly on ash dieback. However, the natural spatial variability of the ash leaf microbiome and the relative contributions of geography, climate, soil conditions, tree characteristics and leaf properties remain insufficiently understood. Examining these relationships is relevant not only in the context of ash dieback but also for developing a general ecological understanding of microbiome assembly in long-lived forest trees exposed to heterogeneous environments.

In this study, we investigated the diversity and composition of bacterial communities associated with leaves from the canopies of *F. excelsior* across geographically and environmentally differing forest sites in Germany. Specifically, we aimed to determine how strongly bacterial communities differ among sites, identify bacterial taxa that are consistently associated with ash across regions, and assess the relative contributions of geographical position, climatic conditions, soil properties, tree characteristics and leaf traits to microbial community variation. Furthermore, we examined microbial association networks as an initial step towards identifying potential interactions among bacterial taxa and determining whether network structures were conserved across sites. Thus, we aimed to answer the following questions: (a) What is the composition of the microbiome of *F. excelsior* and are there significant differences in community composition among sites? (b) Is a core microbiome identifiable across all sites in Germany? (c) Can specific taxa be identified that play a role as hub taxa in microbiome networks? (d) To what degree is the microbiome influenced by biotic and abiotic factors of the sites or of the host tree individuals, and how strong are these effects? Therefore, we sampled and examined 30 ash trees at each of 12 sites across Germany, assessing both their leaf microbiome and the physio-chemical properties of their leaves and the site.

## Methods

### Sites & sampling

**Fig. 1:**
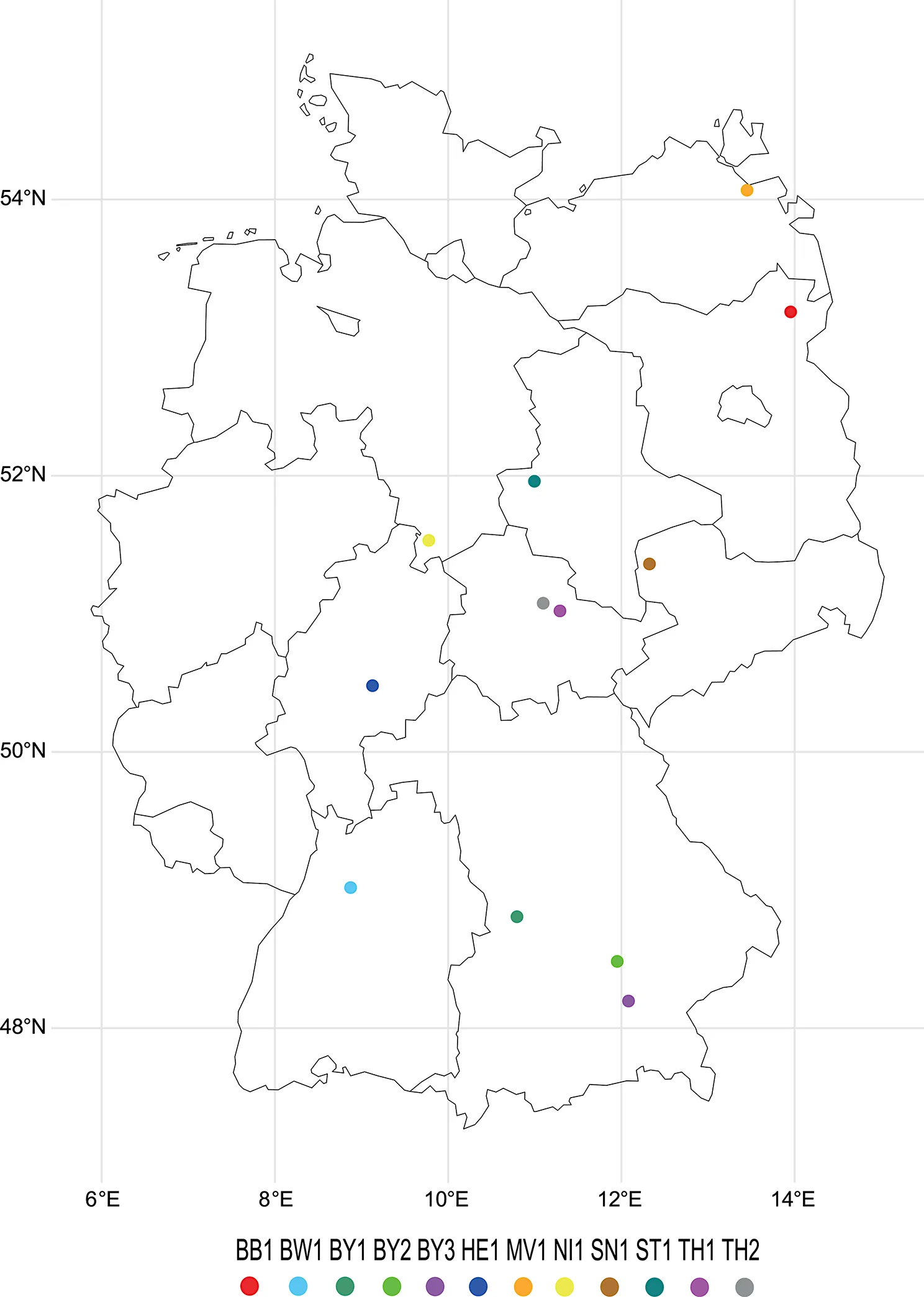
Location of all the sampling sites in Germany; each site is named according to the federal state in which it is located

Within the framework of FraxForFuture, 14 intensive monitoring plots (IBF plots) (Fig. 1) were established [16]. These plots were spread throughout Germany and cover a wide range of ecoregions and typical sites and habitat types of *F. excelsior*, including differences in soil water supply, substrate and covering deposits (Table 1). The plots are located in deciduous, broad-leaved mixed forest with a core area of one hectare [16].

At ten sites, the leaf samples were shot as tufts of leaves with a specially prepared rifle with shot pellets. In MV1, the samples were taken by tree climbers. In SN1, they were taken by slingshot and saw. All methods aimed to take samples from mid-canopy and not close to the stem. The sampling occurred in June 2021 on 12 of the IBF plots. On each plot, leaf samples were taken from 30 trees. Fewer BY3 samples were taken. From each tree, around 20 leaves and petioles of the compound leaves were taken from a mixed sample for microbiome analysis and stored on dry ice for the transport, afterwards at − 20 °C in the freezer until further processing. Additionally, leaves were sampled from the canopy to examine their chemical composition. Mixed leaf samples were immediately frozen on dry ice, stored at − 80 °C, lyophilised, and ground prior to analysis. Following aqueous-methanolic extraction, soluble carbohydrates and several phenolic groups—including total phenols, vanillin-positive compounds, procyanidins, and ortho-dihydroxyphenols—were quantified using photometric assays. Starch was enzymatically determined from the remaining extraction residue, whereas soluble amino acids were analysed chromatographically. Selected individual low-molecular-weight phenolic compounds, including phenolic acids, coumarins, and flavonoids, were additionally characterised using high-performance liquid chromatography (HPLC) [17, 18].

**Table 1:** Information about the sampling sites [16]

Site ID	Site Name	Soil type	Mean Annual Precipitation (mm)	Mean Annual Temperature (°C)	Chest height diameter of mean basal area ash tree [mm]
BB1	Stegelitz, Brandenburg	Gley-parabrown	549	9.1	426
BW1	Plattenwald, Baden-Wuerttemberg	Pelosol-brown	752	10.2	275
BY1	Monheim, Bavaria	Pseudogley-parabrown	759	8.5	386
BY2	Bruckberg, Bavaria	Vega	758	9.3	458
BY3	Isen, Bavaria	Pseudogley colluvisol	994	8.8	424
HE1	Schotten, Hesse	Brown soil pseudogley	934	9	535
MV1	Karlsburg, Mecklenburg- Western Pomerania	Pseudogley-Pararendzina	633	9	637
NI1	Mollenfelde, Lower Saxony	Pelosol	781	8.6	384
SN1	Leutzsch, Saxony	Vega	568	10.2	684
ST1	Huy, Saxony-Anhalt	Terra-Fusca	652	8.9	261
TH1	Ettersberg, Thuringia	Terra-Fusca parabrown	630	8.5	275
TH2	Schwansee, Thuringia	Gley	505	9.8	na*

*na = not available data

### DNA extraction and amplicon sequencing

For DNA extraction, the plant material (2 g of compound leaves) was ground in liquid nitrogen with mortar and pestle. An aliquot of the frozen samples (100 mg) was disrupted again in an MP Fast Prep 24 mill at 5 m*s^− 1^ for 45 s using Lysing Matrix A from MP Biomedicals which also contains a ceramic sphere with a diameter of 6.25 mm. Total DNA was extracted using the DNeasy Plant Mini Kit (Qiagen, Germany). Negative controls without plant material were included during the extraction process. The DNA purity and concentration were measured by a NanoDrop ND-1000 spectrophotometer (Thermo Scientific). For amplicon sequencing, the V5–V6 region of the 16S rRNA genes was amplified by PCR from the DNA samples as described by Ulrich et al. [15] with the bacterial primers 799 F and 1115R, which exclude the chloroplast and mitochondrial DNA of the host plant [19]. PCR amplification was performed in a total reaction volume of 20 µL containing 1–10 ng of template DNA, 15 pmol of each primer, 1.5 U of MyTaq DNA polymerase, 1× MyTaq reaction buffer, and 2 µL of BioStabII PCR Enhancer. The cycling conditions consisted of an initial denaturation at 96 °C for 1 min, followed by 30 cycles of denaturation at 96 °C for 15 s, annealing at 55 °C for 30 s, and extension at 70 °C for 90 s. Amplification of all negative controls did not result in any detectable amplicon. Library preparation and 2 × 300-bp paired-end sequencing on an Illumina MiSeq platform were performed at LGC Genomics (Berlin, Germany).

### Data processing and statistical analyses

The raw sequence data were processed with the DADA2 R package v.1.22 [20]. The algorithm was applied for quality filtering, denoising and removing chimeric sequences. The resulting amplicon sequence variants (ASVs) were taxonomically assigned via the naive Bayesian classifier method using the RDP (ribosomal database project) reference database training set 18. In addition, the assignment was completed at the species level if the ASVs exactly matched the sequence of the reference strain of only one species. After residual plant DNA sequences were removed (0.53% of ASVs) from the dataset, 36.699.819 high-quality bacterial reads were obtained. Statistical analyses were performed using R 4.2.2 [21] with several packages which can be found in Table S3 as well as MicrobiomeAnalyst [22]. All the samples were rarefied to the minimum number of sequences (19468) among all the samples. As result of the dada pipeline and the subsequent rarefying, 321 samples with 126–770 ASVs (mean 314) per sample were obtained. The sample number was BB1 *n* = 26, BW1 *n* = 28, BY1 *n* = 28, BY2 *n* = 25, BY3 *n* = 18, HE1 *n* = 28, MV1 *n* = 29, NI1 *n* = 27, SN1 *n* = 29, ST1 *n* = 27, TH1 *n* = 29, TH2 *n* = 27. Diversity indices, such as richness, Chao 1, Shannon, and Simpson indices, were calculated. The community structure was compared via principal coordinate analysis (PCoA) on the basis of a Bray‒Curtis dissimilarity distance matrix of the ASVs. Significant differences between the communities on the different sites were tested using pairwise comparisons applying analysis of similarity (ANOSIM). To calculate the influence of the environmental and host-related factors on bacterial community composition, a distance-based redundancy analysis (dbRDA) was performed. Beforehand, missing values at the sites were interpolated within the sites. For HE1 and TH2, several factors were missing and could not be interpolated; thus, they were excluded from the dbRDA (*n* = 266). In total, 59 explanatory variables representing geographical position, climate, soil chemistry, tree characteristics, and leaf chemistry (including phenols, procyanidins, and amino acids) were tested. Subsequently, variation partitioning was performed using the significant variables retained in the final dbRDA model to quantify the relative contributions of geographical, climatic, and local soil and host-related factors to the variation in bacterial community composition. To identify bacterial genera with the highest predictive value for site-specific microbiome differentiation, a Random Forest classification model was applied to genus-level relative abundance data. Prior to model fitting, rare genera were removed by retaining only taxa with a mean relative abundance greater than 0.1% and a prevalence of at least 10% across samples. Sampling site was used as the response variable, and genus-level relative abundances were used as predictors. Model performance was evaluated using repeated cross-validation, and the most informative genera were identified based on variable importance scores.

The core microbiome was calculated on the basis of abundance and occurrence [23]. Therefore, genera had to occur in at least 94% of the samples at a site with a relative abundance of > 0.1% to be considered part of the core microbiome of the site. The core microbiome of all the sites was illustrated as a network using Cytoscape version v3.10.1 [24].

In the first approach, a co-occurrence network was constructed at the genus level with a combined dataset of the microbiome from all the sites by applying Sparse Estimation of Correlations among Microbiomes (SECOM) [25] provided by MicrobiomeAnalyst. The correlation threshold was set at 0.3 at a significance level (p value) of 0.05. The network was visualized and analyzed by Cytoscape. The definition of the hub genera was based on Hamonts et al. [26], who revealed the most interactive nodes using the relationships between degree (number of interactions per node), betweenness centrality (fraction of shortest paths the node is on) and closeness centrality (1/[(distance to all other nodes]). The second co-occurrence network was constructed separately for each of the 12 sites. ASVs present in at least 50% of the trees at a site were analyzed using the Molecular Ecological Network Analysis Pipeline [27]. After threshold scanning through the random matrix theory (RMT)-based approach, separate networks were constructed with an identical similarity threshold of 0.60. The topological role of the nodes was assessed by the parameters within-module connectivity (zi) and among-module connectivity (Pi). Connectors (zi ≤ 2.5, Pi > 0.62) are strongly linked to several modules, and module hubs (zi > 2.5, Pi ≤ 0.62) are highly connected to many nodes in their own modules, whereas network hubs (zi > 2.5, Pi > 0.62) act as both module hubs and connectors [27]. The network was also visualized using Cytoscape.

To identify differentially abundant genera at the different sites, the linear discriminant analysis effect size (LEfSe) method was used as provided by MicrobiomeAnalyst. This included the Kruskal‒Wallis test to detect features with significant differences across the groups, followed by pairwise Wilcoxon tests. Finally, linear discriminant analysis (LDA) was applied to estimate the effect size of each genus. Significant genera (FDR < 0.05) with an LDA score greater than 2 were considered most likely to explain the differences between the sampling sites.

## Results

### Microbiome composition across 12 forests in Germany

First, the taxonomic composition of the microbiome of all 321 *F. excelsior* tree leaves was determined which were sampled on the 12 IBF plots. The findings revealed a diverse microbial community, encompassing a total of 26 different phyla. Three phyla—the *Proteobacteria* (correct synonym: *Pseudomonadota*), *Bacteroidetes* (*Bacteroidota*) and *Actinobacteria* (*Actinomycetota*)—consistently dominated across all the sites and samples. *Proteobacteria* was the most abundant phylum, with a mean relative abundance of 58.4% across all the sites. The highest mean relative abundance was observed at SN1 (69.2%), whereas the lowest was shown at BY3 (52.1%). *Bacteroidetes* was the second most abundant phylum, with a mean relative abundance of 21.8% across all the sites. The highest site-level mean relative abundance was observed in TH1 (26.2%), while the lowest was shown at MV1 (18.1%). At the individual sample level, the relative abundance of *Bacteroidetes* ranged from 2.8% at HE1-21 to 51.2% at ST1-25. *Actinobacteria*, the third most abundant phylum, had a mean relative abundance of 16.0% across all sites. Its highest site-level mean abundance was observed at MV1 (22.9%), whereas the lowest was found at SN1 (10.1%). Among individual samples, the relative abundance of *Actinobacteria* ranged from 2.2% (SN1-27) to 44.0% (MV1-15). The remaining 23 phyla collectively accounted for an average relative abundance of 3.8%.

**Fig. 2:**
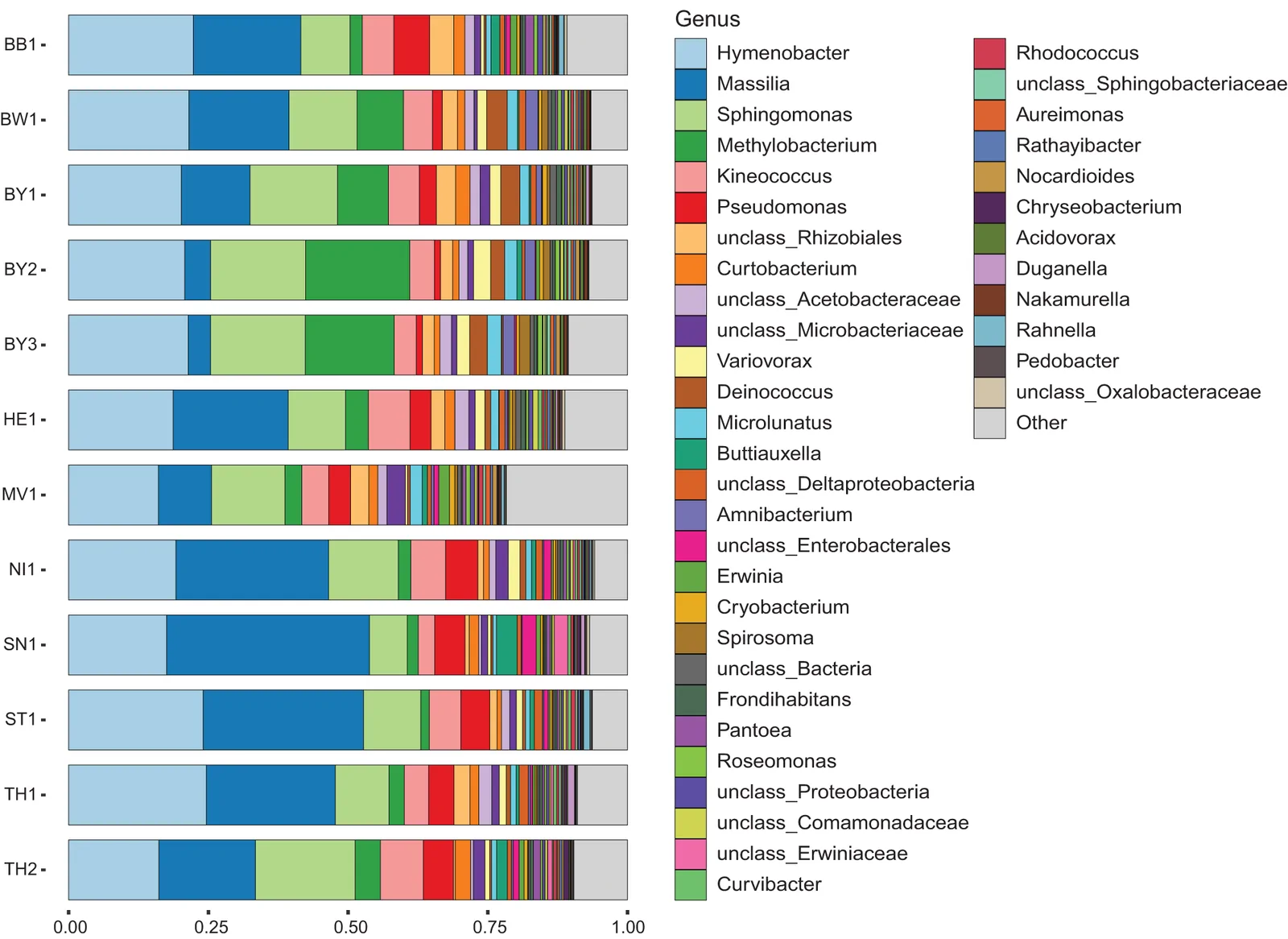
Mean relative abundances of the 40 most abundant bacterial genera in the phyllosphere microbiomes of Fraxinus excelsior, grouped by sampling site. In total, 321 samples (BB1, *n* = 26; BW1, *n* = 28; BY1, *n* = 28; BY2, *n* = 25; BY3, *n* = 18; HE1, *n* = 28; MV1, *n* = 29; NI1, *n* = 27; SN1, *n* = 29; ST1, *n* = 27; TH1, *n* = 29; and TH2, *n* = 27). Bars show the mean relative abundance of each genus across all samples from the respective site. Variability among individual bacterial community profiles was high and is shown in Fig. S1. Error bars are omitted to improve readability. Further information on the sampling sites is provided in Table 1

At the genus level, the microbiome comprised 755 taxa, with substantial variation in community composition among sites. Overall, the three genera *Hymenobacter*, *Massilia* and *Sphingomonas* were the most abundant across all the sites (Fig. 2). However, the relative abundances of these genera varied considerably among both sites and individual samples (Fig. S1). *Hymenobacter* showed a mean relative abundance of 20.2%, with the highest site-level mean abundance at TH1 (24.6%) and the lowest at MV1 (16.1%). At the individual sample level, its relative abundance ranged from 1.8% (HE1-21) to 50.1% (ST1-25). *Massilia* had a mean relative abundance of 18.4%, but its abundance also varied markedly among sites, with the highest site-level mean abundance at SN1 (36.3%) and the lowest at BY3 (4%), Among individual samples, its relative abundance ranged from 0.6% to 66.8%.

**Fig. 3:**
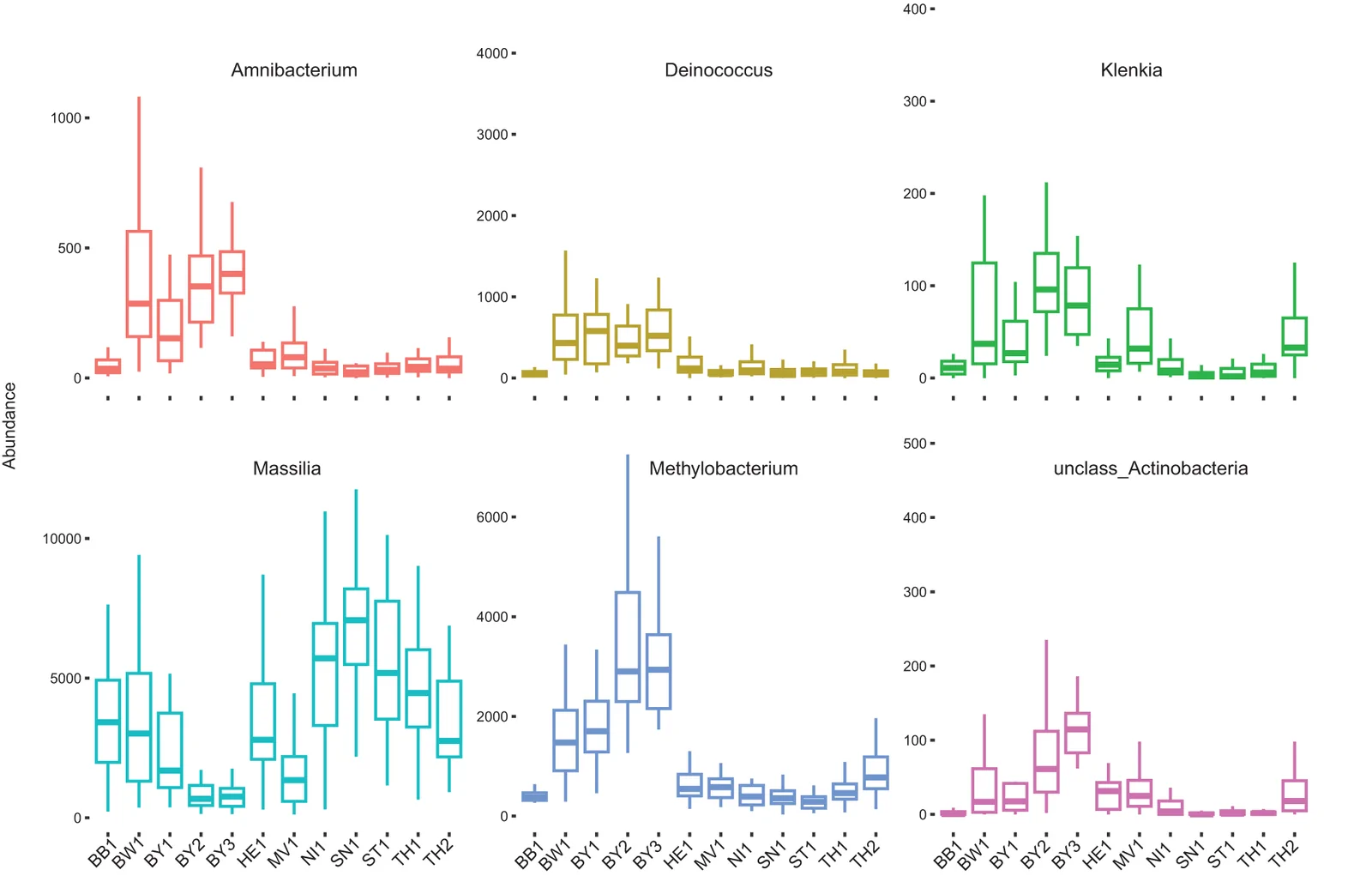
The six genera with the greatest significance in the LEfSe analysis with an average abundance greater than 0.1%

In addition to the overall relevant genera, which are shown in Fig. 2, locally important genera can also be found. MV1 was characterized by comparatively high relative abundances of genera that were not included among the 40 most abundant genera across the complete dataset. For example, the relative abundance of *Aureimonas* reached up to 7.6% in individual MV1 samples, whereas its mean relative abundance across all sites was 0.3%. *Nocardioides* also exhibited comparatively high relative abundances at MV1. Differential abundance analysis was performed using the LEfSe algorithm. The initial statistical analysis identified 87 genera whose relative abundances differed significantly among sites (Kruskal–Wallis test, *p* < 0.01) (Fig. 3). Subsequently, linear discriminant analysis (LDA) was used to estimate the effect size of these differences. Of the 87 significant genera, 21 had LDA scores greater than 2, indicating that they contributed most strongly to the differentiation among sites. These genera included several of the most abundant taxa across the dataset, with *Massilia*, *Methylobacterium*, *Sphingomonas*, *Hymenobacter*, and *Kineococcus* exhibiting the highest LDA scores.

Several of these genera also showed pronounced variation in relative abundance among sites and individual samples. For example, *Methylobacterium* had a mean relative abundance of 6.2% across all sites but reached a site-level mean of 18.6% at BY2. At the individual sample level, its relative abundance ranged from 36.4% to 37.3% in two BY2 samples. Similar site-specific patterns were observed for *Kineococcus* and *Pseudomonas*. *Kineococcus* reached its highest site-level mean relative abundance at TH1 (7.7%), while the highest relative abundance among individual samples was observed in HE1-14 (21.4%). Likewise, although *Pseudomonas* had a mean relative abundance of 3.9% across all sites, it reached 25.2% in sample BY1-25.

Additionally, a Random Forest classification based on genus-level relative abundances further demonstrated the site specificity of the bacterial communities. The model achieved a cross-validated accuracy of 62.8% (Kappa = 0.59), while the final model showed an out-of-bag classification accuracy of 59.5%. Classification success varied among sites, with samples from SN1 (89.7%), MV1 (82.8%), TH2 (77.8%), and BY2 (76%) being assigned most accurately, whereas TH1 (31%), ST1 (37%), and NI1 (37%) exhibited the highest rates of misclassification. Misclassifications occurred particularly between BY2 and BY3 as well as between ST1 and TH1. The genera with the highest predictive value for site classification included *Methylobacterium*, *Diplorickettsia*, *Buttiauxella*, *Klenkia*, *Massilia*, *Nocardioides*, *Sphingomonas*, and *Aureimonas*. Several of these genera, including *Methylobacterium*, *Massilia*, and *Sphingomonas*, were also identified by LEfSe/LDA as differentially abundant among sites, highlighting their importance for geographical differentiation of the ash phyllosphere microbiome.

In the next step, the alpha diversity of the samples and sites was estimated at the ASV level, using richness, Chao1, Shannon and Simpson indices (Table S1). All the indices differed significantly across the sites. Chao1 diversity was highest at BY3 (540 ± 166) and was significantly greater than that at the other sites, execpt MV1 (434 ± 147), HE1 (383 ± 120) and BY2 (408 ± 159). These three sites did not differ significantly from that at BY3. Although, MV1, HE1 and BY2 showed significantly greater diversity than SN1 (288 ± 138), ST1 (245 ± 73.2) and TH1 (274 ± 68.5). The observed richness exhibited a similar pattern, as the richness at BY3 (485 ± 142) was significantly more diverse than that at eight other sites but did not differ from that at BY2 (369 ± 135), HE1 (357 ± 105) and MV1 (408 ± 131). Only the diversity of MV1 was significantly higher than that of BW1 (284 ± 90.4), NI1 (287 ± 70.1) and the eastern sites SN1 (262 ± 124), ST1 (231 ± 64.4), TH 1 (254 ± 55.1) and TH2 (283 ± 120). The Shannon Index showed fewer significant differences, but MV1 (4.29 ± 0.52) was significantly more diverse than six other sites. The Simpson Index showed few differences, but different from the other indices. SN1 (0.956 ± 0.036) was significantly more diverse compared with BY1 (0.947 ± 0.021) and TH1 (0.948 ± 0.019). In addition, TH2 (0.963 ± 0.009) was significantly more diverse than TH1.

Additionally, a PCoA was performed based on Bray‒Curtis dissimilarity to determine differences in the communities between the different sites at the ASV level. The first two axes of the PCoA plot explain 13.3% and 6.3% of the total variance (Fig. S2). The BY3 and BY2 samples are separated from the other sites to a certain extent. MV1 is wider spread but mostly separated from the other sides. BY1, BW1 and HE1 overlap mostly with each other and BY2. The other sites mostly overlap. However, ANOSIM revealed that the sites generally differed significantly from each other (*R* = 0.42, *p* = 0.001). On the basis of pairwise ANOSIM, most of the sites were also significantly different from the others (*p* < 0.001, Table S1), with the greatest difference between BY3 and many of the other sites. Between the NI1 and ST1 and TH1 sites, as well as between ST1 and TH2, the differences were less significant. No significant difference was found between ST1 and TH1 (*p* > 0.05).

### Biotic and abiotic drivers

**Fig. 4:**
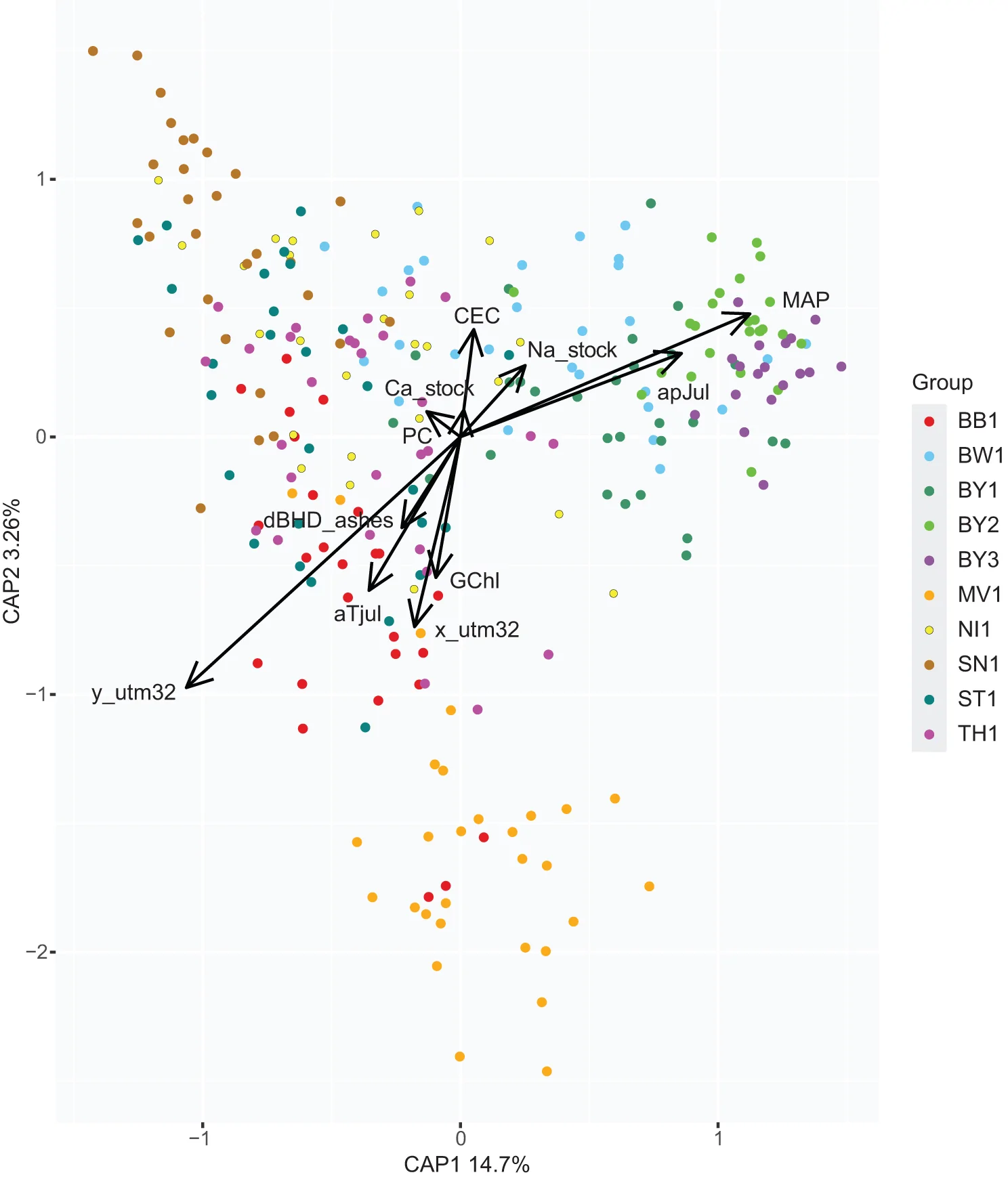
Distance-based redundancy analysis (dbRDA) showing the relationships between community composition and environmental variables at ten sites along the first to constrained axes. The arrows represent environmental variables, with their length and direction indicating the strength and direction of influence on community composition. apJul = average precipitation in July, aTjul = average temperature in July, Ca_stock = calcium stock in the soil, CAC = cation exchange capacity, dBHD_ashes = average chest height diameter of the ash trees, GChl = chlorophyll in the ash leaves, MAP = mean annual precipitation, Na_stock = amount of sodium in the soil, PC = phytochelatine, x_utm32 = west‒east position within Germany, y_utm32 = north‒south position within Germany Samples are plotted on the basis of their ordination scores

We performed distance-based redundancy analysis (dbRDA) to examine the relationships between environmental variables and microbiome composition (Fig. 4). The first two axes of the dbRDA explained 14.7% (CAP1) and 3.26% (CAP2) of the total variation, respectively. The samples from sites BY2 and BY3 were separated from the other samples. Most of the MV1 samples were also distinct from the samples from the other sites and only slightly overlapped. Additionally, the samples of SN1 were partly separated from the main group. The analysis revealed that geographical position, precipitation, tree age and the leaf and soil chemistry were significant factors that explained the variance. Together, these factors explain 28% of variance. The length and direction of the vectors show that the mean annual precipitation and the average precipitation in July were drivers of the community composition mainly along CAP1 and were strongly associated with BY2 and BY3. Cation exchange capacity, geographical east value, average temperature in July, total chlorophyll content and average chest height diameter of the ashes are the main contributors to the variation along CAP2. Factors such as the geographical north value, the sodium stock and the calcium stock explain the variation along both CAPs. However, as mentioned above, the factors given do not explain all the variance of the samples. The greatest influence was shown by the geographical north value, which indicates a general shift in the microbiome from north to south. The second greatest influence is the mean annual precipitation, which is negatively correlated with the geographical north value and is close to the plots in southern Germany.

To quantify the relative importance of the significant explanatory variables, variation partitioning was performed using the variables retained in the dbRDA. Geographical position, climate, and local soil and host-related variables together explained 22.1% of the total variation in bacterial community composition (adjusted R²). Climate (6.7%) and local soil and host-related variables (6.6%) accounted for the largest independent fractions of explained variation, whereas geographical position alone explained only 0.8%. However, geography shared a substantial fraction of explained variation (7.9%) with climate and local environmental variables, indicating that spatial differences in microbiome composition were largely associated with underlying climatic and environmental gradients rather than geographical distance itself. The remaining 77.9% of the variation remained unexplained.

### Core microbiome

**Fig. 5:**
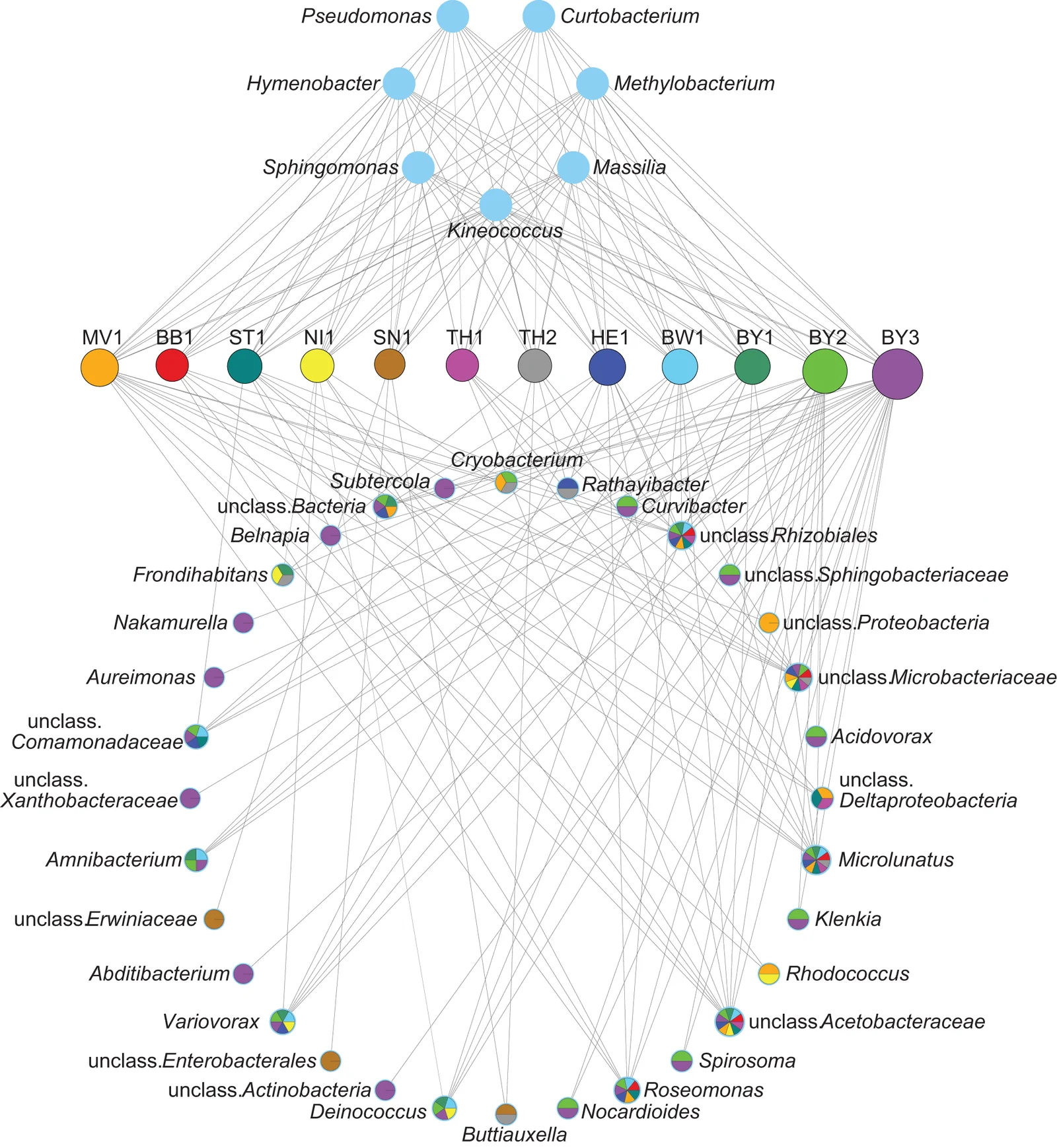
Core microbiome of *F. excelsior* at the genus level. The core microbiome was defined as containing taxa that were present in at least 94% of the samples at one site and whose relative abundance was at least 0.1%. The seven genera shared by all the sites are shown on top. Under the sites, which are ordered from north to south, the other genera of the core microbiomes are presented. The colors indicate the sites on which the genera are part of the core microbiome. The node size of the sites reflects the number of genera of the microbiome

In the next step, the core microbiome of *F. excelsior* was determined at the genus level. In total, 38 genera were among the different core microbiomes in the plots (Fig. 5). SN1 had the lowest number of genera in the core microbiome (10), whereas BY3 had the greatest number of genera (30). Seven genera were detected in the core microbiomes of all the plots, including genera such as *Hymenobacter* and *Sphingomonas*, whose relative abundances were relatively high, and *Curtobacterium* and *Pseudomonas*, whose relative abundances were relatively low. Several genera can be found in the core microbiome of most plots, such as *Microlunatus* (10 sites) and *Roseomonas* (7 sites). Furthermore, some genera were specific to one plot or could be found only on plots of the same geographical region. In particular, BY2 and BY3 were sticking out and uniquely shared genera such as *Curvibacter*, *Acidovorax* and *Klenkia*. Only two plots contained genera that are specific for their core microbiome. An unclassified *Enterobacterales* genus and an unclassified *Erwiniaceae* genus occurred only in SN1. BY3 was sticking out with seven specific genera, e.g., *Aureimonas*, *Nakamurella* and *Subtercola*.

### Network analysis

**Fig. 6:**
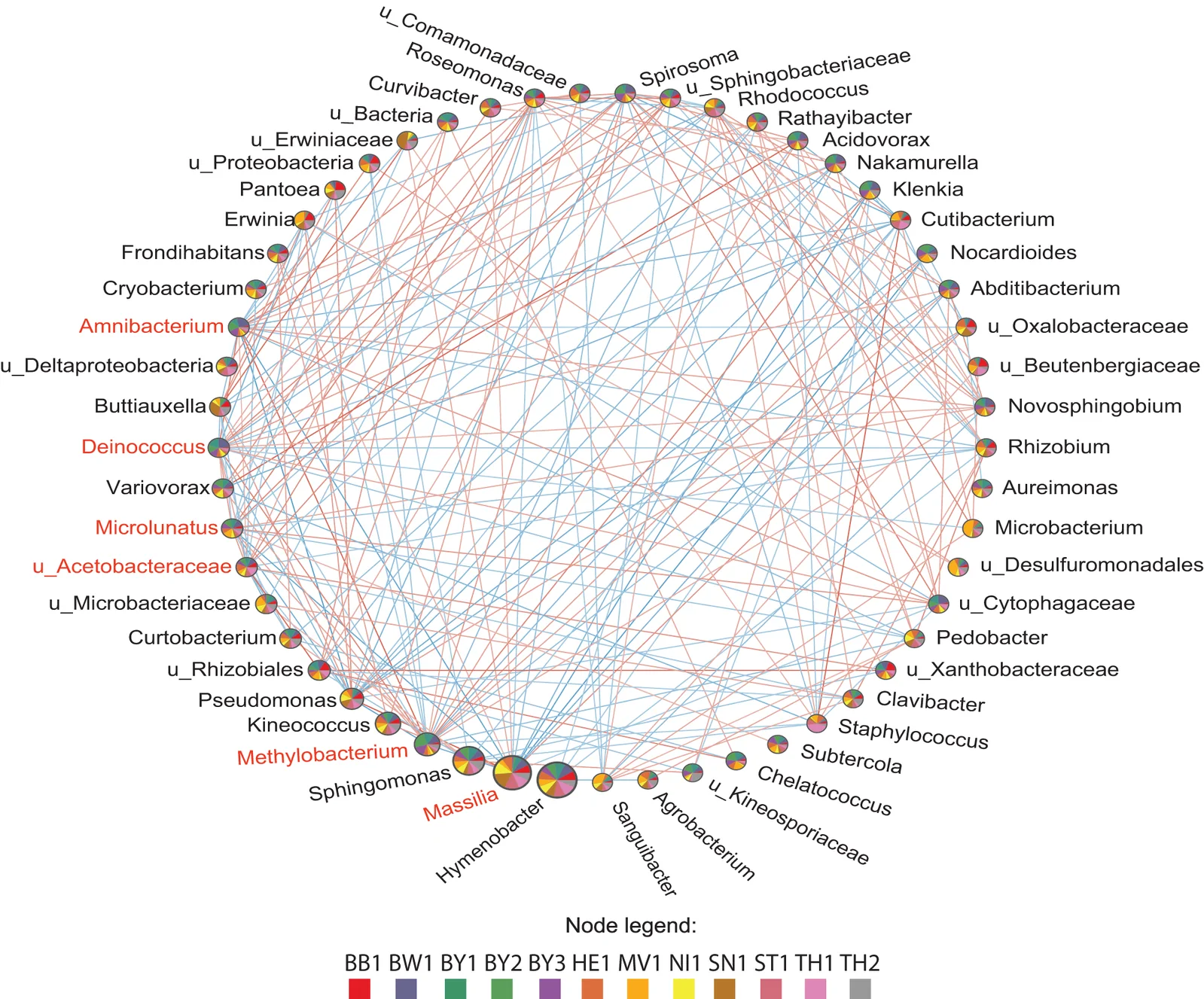
Network analysis at the genus level of *F. excelsior* showing at least one significant connection to another genus. The edges show negative (blue) or positive (red) correlations between the genera. The size of the nodes represents the relative abundance of the genera, while the color indicates the distribution at the different sites. Red names indicate hub genera

A comprehensive analysis of the microbiome for all the sites revealed a complex co-occurrence network with a total of 53 genera (nodes) and 224 edges (number of associations) (Fig. 6). The network density was 0.163. On the basis of the analysis of betweenness and closeness centrality and the degree (number of associations per node), six hub genera were identified: *Amnibacterium*, *Deinococcus*, *Microlunatus*, unclassified *Acetobacteraceae*, *Methylobacterium*, and *Massilia*. *Methylobacterium* and *Deinococcus* exhibited the highest degree of connectivity, each forming 22 edges with other nodes. In contrast, *Aureimonas*, *Subtercola*, and an unclassified genus within *Deltaproteobacteria* were the least connected, with only one edge each. The correlations within the network included 116 positive correlations and 108 negative correlations. The strongest positive correlation was observed between *Pseudomonas* and *Massilia*, and the strongest negative correlation was found between *Pseudomonas* and the hub genus *Amnibacterium*.

**Fig. 7:**
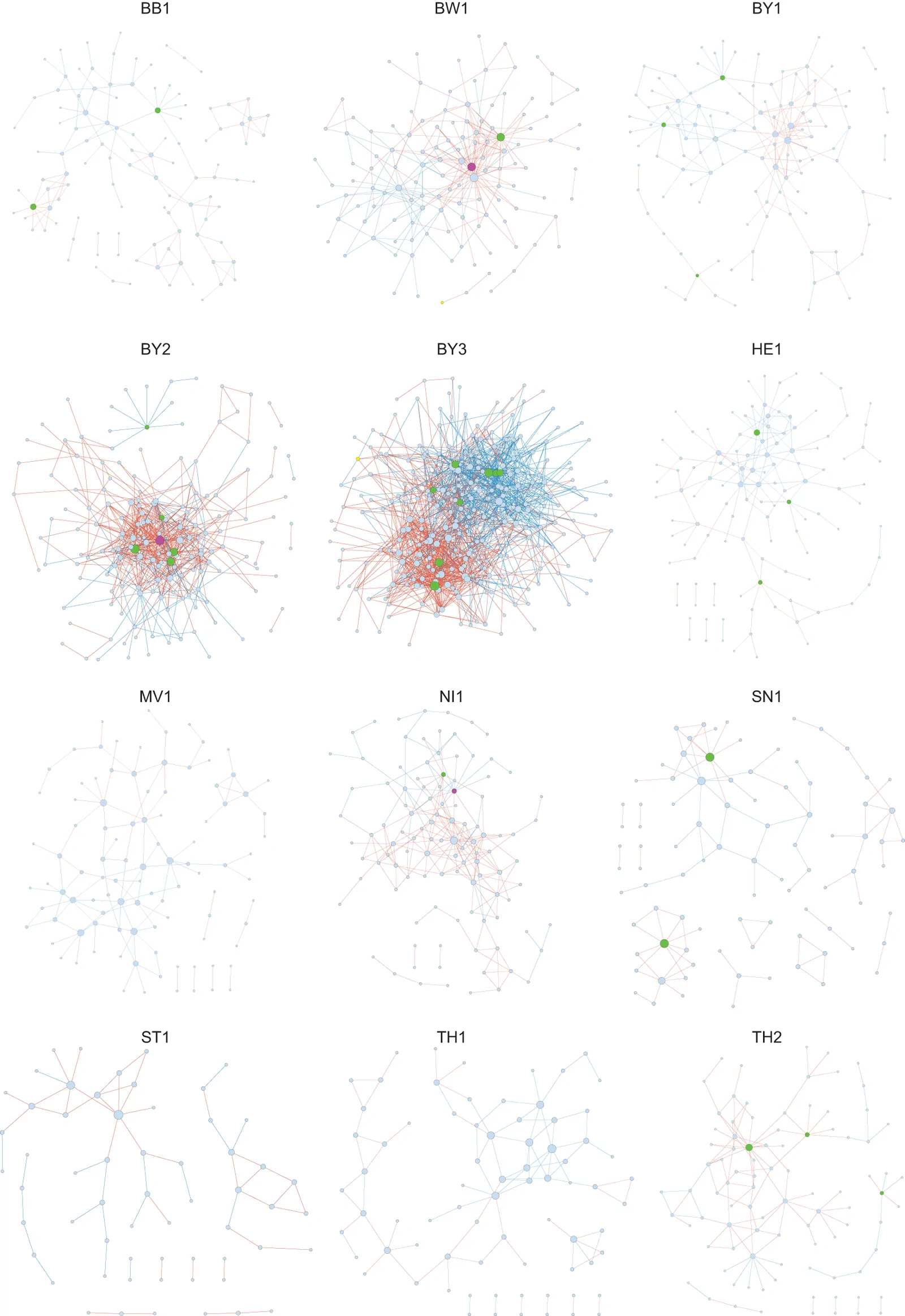
Network analysis at the OTU level of the *F. excelsior* microbiome for the different sampling sites. The edges indicate negative (blue) or positive (red) correlations between the OTUs. The size of the nodes reflects the number of connections. The green nodes represent modular hubs, and the pink nodes represent network hubs

Additionally, a network analysis was conducted separately at the ASV level to identify interactions between the members of the microbiome at each site. The sites clearly differed in terms of network complexity (Fig. 7). The sites in southern Germany (BW1, BY1-3) were more complex compared with the other sites and contained two of the three network hubs and 17 of 28 modular hubs. BY3 is noteworthy and is the most complex network, with 294 nodes and 1342 edges. Interestingly, compared with the southern German sites, NI1, with 108 notes and 213 edges, is less complex but nevertheless has a centralized network with one network hub, a modular hub and the highest network density. Three sites have no hubs, of which ST1 and TH1 are also apparently the least complex networks.

## Discussion

The phyllosphere comprises the aerial parts of plants, mostly the leaves. They are densely colonized by Bacteria on their surface as epiphytes, but also inside as endophytes [28]. Across diverse plant hosts, including *Arabidopsis thaliana*, *Citrus* spp., and *Triticum aestivum*, bacterial communities are frequently dominated by *Proteobacteria*, *Bacteroidetes*, *Actinobacteria*, and *Firmicutes* [6, 28, 29]. Similar phyla are commonly detected in tree canopies, although their relative abundances vary among host species. For example, *Proteobacteria* dominate the phyllosphere communities of *Betula pendula* and *Quercus robur*, whereas *Firmicutes* were reported to be more abundant on *Pinus sylvestris* [30].

In response to the spread of ash dieback in the late 20th century, increasing attention has been directed towards the leaf microbiome of *Fraxinus excelsior*. Previous studies identified *Proteobacteria*, *Bacteroidetes*,* Actinobacteria* and *Deinococcus*-*Thermus* as the most abundant phyla associated with ash leaves [13, 15], which is consistent with our results. At the genus level, earlier studies reported high abundances of *Sphingomonas*, *Hymenobacter*, *Methylobacterium*, and *Pseudomonas* [15]. Although community composition varied strongly among individual samples and sites in our study, the communities were generally dominated by *Hymenobacter*, *Massilia*, *Sphingomonas*, and *Methylobacterium*. Thus, our findings confirm the prominence of several genera previously associated with the ash phyllosphere, while also identifying *Massilia* as an abundant component of the communities examined here. All four genera were also members of the core microbiome shared across the investigated sites.

None of these dominant genera exist exclusively in the ash microbiome. *Methylobacterium* and *Sphingomonas* are widely distributed in plant-associated microbiomes [6, 31], while *Hymenobacter*, *Massilia*, and *Sphingomonas* have also been reported as dominant members of the phyllosphere of *Prunus* spp. Janakiev et al. [32]. *Massilia* is frequently detected in soil communities but has also been found in the leaf microbiome of *A. thaliana* [33, 34] The repeated occurrence of these genera across diverse hosts suggests that they possess traits favouring colonisation of aerial plant habitats. In particular, their tolerance of nutrient limitation, radiation, and intermittent desiccation may contribute to their persistence in tree canopies [35]. The consistent detection of *Hymenobacter*, *Massilia*, *Sphingomonas*, and *Methylobacterium* across all sites is therefore compatible with broad adaptation to phyllosphere conditions.

Co-occurrence network analysis provided an additional perspective on community organisation by identifying taxa with numerous statistical associations, although these associations cannot reveal the specific nature of the interactions [3, 36]. Despite its low relative abundance, *Deinococcus*, exhibited high connectivity and was identified as a network hub. *Deinococcus* spp. are highly resistant to radiation, oxidative stress and desiccation [37]. This gives the members of this genus advantages on the surfaces of leaves, as these are habitats of high radiation and water scarcity. The ability to form a biofilm is of advantage as it may provide space and physically stable boundary conditions for other bacteria, making *Deinococcus* a pivotal member of the ash microbiome. *Methylobacterium* is ubiquitously found in the phyllosphere. It grows on methanol, a volatile organic compound produced by the plant, and can produce plant growth-promoting substances such as cytokinins, auxins, and vitamin B_12_ [38, 39]. Overall, the core shared among all sites consisted primarily of genera that are widespread across plant species and may be broadly adapted to phyllosphere conditions. In contrast, differences in the site-specific core communities suggest that local environmental conditions, host characteristics, dispersal processes, or other site-level factors contribute to regional community differentiation.

We aimed to determine which host-related and environmental factors contribute to shaping the ash phyllosphere microbiome. Microorganisms inhabiting exposed leaf surfaces experience harsh conditions such as fluctuating temperatures, radiation, intermittent desiccation, and limited nutrient availability, resulting in strong environmental filtering of phyllosphere communities [35, 40]. The factors shaping these communities can be categorized into 4 categories: host identity, season, geographical location and anthropogenic influences such pollutants [6]. The present study focused primarily on intraspecific host characteristics and site-level environmental conditions.

Host species identity (i.e. tree species and genotype) is frequently reported as a stronger determinant of temperate tree phyllosphere bacterial communities than sampling site or time [6, 41, 42]. Because only *Fraxinus excelsior* was investigated here, variation within our dataset is more likely to reflect differences among individual trees in their genetic background, developmental stage, physiological condition, and leaf chemistry. Previous studies have identified differences between the microbiomes of ash trees showing contrasting susceptibility to *Hymenoscyphus fraxineus* [15]. and microbiome differentiation has also been observed among ash seedlings associated with pathogen-tolerant parent trees [43]. These findings indicate that intraspecific host characteristics may contribute to phyllosphere community assembly.

In our study, ash diameter at breast height was significantly associated with bacterial community composition. This relationship suggests a possible effect of tree size or developmental stage, consistent with previous reports that host age and leaf development influence plant-associated microbiomes [44, 45]. However, DBH is also affected by stand structure, growth conditions, and management history and should therefore be regarded only as an approximate proxy for age. Chlorophyll and procyanidin contents were also associated with community composition. Procyanidins and other phenolic compounds may influence microbial colonisation through their antimicrobial or signaling properties [46], whereas chlorophyll content may reflect variation in host physiological status, nutrient availability, leaf development, or responses to environmental stress [47]. These physiological responses of the host plant to stressors can also lead to changes in microbiome composition [29, 48]. These relationships support an association between host condition, leaf chemistry, and microbiome structure. As the host genotype was not assessed, unmeasured genetic variation may explain part of the remaining community variation [44].

In addition to the host-related factors, bacterial community composition differed significantly among sampling sites and was associated with geographical factors particularly along the north–south gradient. However, variance partitioning showed that the fraction explained exclusively by geography was small, whereas a larger proportion of community variation was jointly explained by geography and climate. This indicates that the observed spatial pattern was primarily associated with climatic gradients that covaried with geographical position rather than with geographical distance alone. Temperature and precipitation were also significantly associated with community composition, consistent with previous evidence that climatic conditions affect both epiphytic and endophytic microorganisms [49]. Although dispersal limitation, shared regional microbial pools, and other spatially structured factors may still contribute to differences among sites [6, 50, 51], as the small fraction of variation explained exclusively by geographical position suggests. that their independent contribution was limited. But the shared fractions identified by variance partitioning further indicate that the effects of geography and climate could not be completely separated.

Soil properties were also associated with variation in the phyllosphere microbiome, although a direct connection between soil properties and the phyllosphere is difficult to draw. Previous studies have indicated that soil properties can indirectly influence phyllosphere fungal diversity potentially through effects on host nutrition, physiology, and leaf chemistry [52]. In our analysis, soil Ca and Na stocks and cation-exchange capacity were significantly associated with community composition. These variables may influence phyllosphere bacteria indirectly through changes in nutrient availability and host phenotype, but they may also represent broader differences among sites. Overall, the results suggest that the ash phyllosphere microbiome is structured by the combined and partly overlapping effects of host characteristics, climate, geographical position, and soil and leaf chemistry. The pronounced differences among sites therefore appear to reflect the specific combination of environmental and host-related conditions at each location rather than geographical position alone. Together, the measured factors explained 22% of the variation in bacterial community composition, suggesting that additional variables, including host genotype, microenvironmental conditions, dispersal, temporal dynamics, and stochastic processes, contribute to community assembly. Because leaf metabolite profiles are dynamic phenotypic traits influenced by both genetic and environmental conditions, they cannot substitute for direct information on host genotype.

## Conclusions

Forest ecosystems are under pressure from climate change and diseases We investigated the diversity of the leaf microbiome of *F. excelsior* and how the geographic position as well as abiotic and individual host factors affect it. We proved that the ash phyllosphere microbiome is generally quite variable but dominated by few bacterial genera - *Hymenobacter*, *Massilia* and *Sphingomonas*. A ash-specific core microbiome was identified comprising seven genera present at all the sites and trees being investigated. Each site additionally contained genera which formed a bigger locally different core microbiome. The network analyses revealed that less frequent genera play important roles as hub species. Even when analyzed separately by site, the local networks showed differences in connectivity and density. We were also able to show the modulating role of host-related and environmental factors, indicating that spatial differences in microbiome composition were largely associated with underlying climatic and environmental gradients rather than geographical distance itself.

Considering the potential of using the microbiome or specific members as biocontrol agents against ash dieback and its causal agent, it is crucial to account for regional factors to ensure successful establishment of the introduced microorganisms on the host. As a next step the role of the genotype of the ash trees as well as the different strata in the canopy for their role in shaping the phyllosphere microbiome as well its dynamics over the year should be investigated to further establish which bacterial taxa are crucial for a stable, protective and beneficial ash leaf microbiome.

## Supplementary Information

Below is the link to the electronic supplementary material.

Supplementary Material 1

Supplementary Material 2

Supplementary Material 3

## Data Availability

The paired sequence reads generated for the microbiome analysis were deposited in the NCBI Sequence Read Archive under Accession Number PRJNA1087259. The R scripts are archived as a GitHub release: https://github.com/vburg/ash-microbiome-and-its-modulating-factors.

## References

[1] Forest Europe. State of Europe's forests 2020 [Internet]. 2020 [cited 2025 Jul 30]. Available from: https://foresteurope.org/state-of-europes-forests/

[2] Berg G, Dorador C, Egamberdieva D, Kostka JE, Ryu C-M, Wassermann B. Shared governance in the plant holobiont and implications for One Health. FEMS Microbiol Ecol. 2024;100(3). 10.1093/femsec/fiae004. PMC10876113 38364305

[3] Berg G, Rybakova D, Fischer D, Cernava T, Vergès M-CC, Charles T, Chen X, et al. Microbiome definition re-visited: old concepts and new challenges. Microbiome. 2020;8(1):103. 10.1186/s40168-020-00875-0. PMC7329523 32605663

[4] Compant S, Samad A, Faist H, Sessitsch A. A review on the plant microbiome: Ecology, functions, and emerging trends in microbial application. J Adv Res. 2019;19:29–37. 10.1016/j.jare.2019.03.004. PMC6630030 31341667

[5] Leveau JHJ. A brief from the leaf: latest research to inform our understanding of the phyllosphere microbiome. Curr Opin Microbiol. 2019;49:41–9. 10.1016/j.mib.2019.10.002. 31707206

[6] Bashir I, War AF, Rafiq I, Reshi ZA, Rashid I, Shouche YS. Phyllosphere microbiome: Diversity and functions. Microbiol Res. 2022;254:126888. 10.1016/j.micres.2021.126888. 34700185

[7] Liu H, Brettell LE, Singh B. Linking the Phyllosphere Microbiome to Plant Health. Trends Plant Sci. 2020;25(9):841–4. 10.1016/j.tplants.2020.06.003. 32576433

[8] Pieterse CM, Zamioudis C, Berendsen RL, Weller DM, Van Wees SC, Bakker PA. Induced systemic resistance by beneficial microbes. Annu Rev Phytopathol. 2014;52:347–75. 10.1146/annurev-phyto-082712-102340. 24906124

[9] Romera FJ, Garcia MJ, Lucena C, Martinez-Medina A, Aparicio MA, Ramos J, Alcantara E, et al. Induced systemic resistance (ISR) and Fe deficiency responses in dicot plants. Front Plant Sci. 2019;10:287. 10.3389/fpls.2019.00287. PMC6421314 30915094

[10] Moënne-Loccoz Y, Mavingui P, Combes C, Normand P, Steinberg C Microorganisms and biotic interactions. In: Bertrand JC, Caumette P, Lebaron P, Matheron R, Normand P, Sime-Ngando T, editors. Environmental microbiology: fundamentals and applications: microbial ecology. Dordrecht: Springer Netherlands; 2015. p. 395-444.

[11] McLaren MR, Callahan BJ. Pathogen resistance may be the principal evolutionary advantage provided by the microbiome. Philos Trans R Soc Lond B Biol Sci. 2020;375(1808):20190592. 10.1098/rstb.2019.0592. PMC7435163 32772671

[12] Lefevere H, Bauters L, Gheysen G. Salicylic acid biosynthesis in plants. [Mini Review]. 2020;11(338). 10.3389/fpls.2020.00338. PMC7182001 32362901

[13] Griffiths SM, Galambao M, Rowntree J, Goodhead I, Hall J, O’Brien D, Atkinson N, et al. Complex associations between cross-kingdom microbial endophytes and host genotype in ash dieback disease dynamics. J Ecol. 2019;108(1):291–309. 10.1111/1365-2745.13302.

[14] Becker R, Ulrich K, Behrendt U, Kube M, Ulrich A. Analyzing ash leaf-colonizing fungal communities for their biological control of *Hymenoscyphus fraxineus*. [Original Research]. Front Microbiol. 2020;11(2653):590944. 10.3389/fmicb.2020.590944. PMC7649789 33193255

[15] Ulrich K, Becker R, Behrendt U, Kube M, Ulrich A. A comparative analysis of ash leaf-colonizing bacterial communities identifies putative antagonists of *Hymenoscyphus fraxineus*. Front Microbiol. 2020;11:966. 10.3389/fmicb.2020.00966. PMC7273808 32547506

[16] Langer GJ, Fuchs S, Osewold J, Peters S, Schrewe F, Ridley M, Kätzel R, et al. FraxForFuture—research on European ash dieback in Germany. J Plant Dis Prot. 2022;129(6):1285–95. 10.1007/s41348-022-00670-z.

[17] Häuser H, Hauswald J, Kätzel R. Comparative studies on the identification and assessment of basal stem necrosis in ash trees (Fraxinus excelsior) against the backdrop of the European ash dieback. J Plant Dis Prot. 2025a;132(5):147. 10.1007/s41348-025-01126-w.

[18] Häuser H, Pilger A, Ulrichs C, Kätzel R. Phenolic Leaf Compounds in Ash Trees (Fraxinus excelsior L.) in the Context of Ash Dieback. Forests. 2025b;16(9):1387.

[19] Laforest-Lapointe I, Paquette A, Messier C, Kembel SW. Leaf bacterial diversity mediates plant diversity and ecosystem function relationships. Nature. 2017;546(7656):145–7. 10.1038/nature22399. 28538736

[20] CallahanBJ, McMurdie, PJ, Rosen, MJ, Han, AW, Johnson, AJ, & Holmes, SP (2016).DADA2: High-resolution sample inference from Illumina amplicon data. Nat Methods, 13(7), 581-583. 10.1038/nmeth.3869 PMC4927377 27214047

[21] R Core Team(2023). R: A language and environment for statistical computing. R Foundation for Statistical Computing. (4.2.2 ed.). https://www.R-project.org/

[22] Lu Y, Zhou,G, Ewald, J, Pang, Z, Shiri, T, & Xia, J (2023). MicrobiomeAnalyst 2.0: comprehensive statistical, functional and integrative analysis of microbiomedata. Nucleic Acids Res, 51(W1), W310-W318. 10.1093/nar/gkad407 PMC10320150 37166960

[23] Neu AT, Allen EE, Roy K. Defining and quantifying the core microbiome: Challenges and prospects. Proc Natl Acad Sci. 2021;118(51):e2104429118. 10.1073/pnas.2104429118. PMC8713806 34862327

[24] Shannon P, Markiel A, Ozier O, Baliga NS, Wang JT, Ramage D, Amin N, et al. Cytoscape: a software environment for integrated models of biomolecular interaction networks. Genome Res. 2003;13(11):2498–504. 10.1101/gr.1239303. PMC403769 14597658

[25] Lin H, Eggesbø M, Peddada SD. Linear and nonlinear correlation estimators unveil undescribed taxa interactions in microbiome data. Nat Commun. 2022;13(1):4946. 10.1038/s41467-022-32243-x. PMC9399263 35999204

[26] Hamonts K, Trivedi P, Garg A, Janitz C, Grinyer J, Holford P, Botha FC, et al. Field study reveals core plant microbiota and relative importance of their drivers. Environ Microbio. 2018;20(1):124–40. 10.1111/1462-2920.14031. 29266641

[27] Deng Y, Jiang Y-H, Yang Y, He Z, Luo F, Zhou J. Molecular ecological network analyses. BMC Bioinform. 2012;13(1):113. 10.1186/1471-2105-13-113. PMC3428680 22646978

[28] Schlechter RO, Miebach M, Remus-Emsermann MNP. Driving factors of epiphytic bacterial communities: A review. J Adv Res. 2019;19:57–65. 10.1016/j.jare.2019.03.003. PMC6630024 31341670

[29] Francioli D, Cid G, Hajirezaei M-R, Kolb S. Leaf bacterial microbiota response to flooding is controlled by plant phenology in wheat (Triticum aestivum L). Sci Rep. 2022a;12(1):11197. 10.1038/s41598-022-15133-6. PMC9249782 35778470

[30] Duan Y, Siegenthaler A, Skidmore AK, Chariton AA, Laros I, Rousseau M, De Groot GA. Forest top canopy bacterial communities are influenced by elevation and host tree traits. Environ Microbiome. 2024;19(1):21. 10.1186/s40793-024-00565-6. PMC10998314 38581032

[31] Delmotte N, Knief C, Chaffron S, Innerebner G, Roschitzki B, Schlapbach R, von Mering C, Vorholt JA. Community proteogenomics reveals insights into the physiology of phyllosphere bacteria. Proc Natl Acad Sci. 2009;106(38):16428–33. 10.1073/pnas.0905240106. PMC2738620 19805315

[32] Janakiev T, Berić T, Stević T, Stanković S, Bačić J, Majstorović H, Fira D, Dimkić I. The Microbiome of the ‘Williams’ pear variety grown in the organic orchard and antifungal activity by the autochthonous bacterial and yeast isolates. Microorganisms. 2022;10(7). 10.3390/microorganisms10071282. PMC9321879 35889000

[33] Bodenhausen N, Horton MW, Bergelson J. Bacterial Communities Associated with the Leaves and the Roots of Arabidopsis thaliana. PLoS ONE. 2013;8(2):e56329. 10.1371/journal.pone.0056329. PMC3574144 23457551

[34] Xu A, Liu C, Zhao S, Song Z, Sun H. Dynamic distribution of massilia spp. in sewage, substrate, plant rhizosphere/phyllosphere and air of constructed wetland ecosystem. Front Microbiol. 2023;14. 10.3389/fmicb.2023.1211649. PMC10413979 37577432

[35] Herrmann M, Geesink P, Richter R, Küsel K. Canopy Position Has a Stronger Effect than Tree Species Identity on Phyllosphere Bacterial Diversity in a Floodplain Hardwood Forest. Microb Ecol. 2021;81(1):157–68. 10.1007/s00248-020-01565-y. PMC7794210 32761502

[36] Barberán A, Bates ST, Casamayor EO, Fierer N. Using network analysis to explore co-occurrence patterns in soil microbial communities. ISME J. 2011;6(2):343–51. 10.1038/ismej.2011.119. PMC3260507 21900968

[37] Gerber E, Bernard R, Castang S, Chabot N, Coze F, Dreux-Zigha A, Hauser E, et al. Deinococcus as new chassis for industrial biotechnology: biology, physiology and tools. J Appl Microbiol. 2015;119(1):1–10. 10.1111/jam.12808. PMC4682472 25809882

[38] Jahan R, McDonald IR. Diversity of Methylobacterium species associated with New Zealand native plants. FEMS Microbiol Lett. 2023;370. 10.1093/femsle/fnad124. PMC10699869 37985695

[39] Kanukollu S, Remus R, Rücker AM, Buchen-Tschiskale C, Hoffmann M, Kolb S. Methanol utilizers of the rhizosphere and phyllosphere of a common grass and forb host species. Environ Microbiome. 2022;17(1):35. 10.1186/s40793-022-00428-y. PMC9258066 35794633

[40] Chun S-J, Cui Y, Yoo S-H, Lee JR. Organic connection of holobiont components and the essential roles of core microbes in the holobiont formation of feral brassica napus. Front Microbiol. 2022;13. 10.3389/fmicb.2022.920759. PMC9305074 35875588

[41] Laforest-Lapointe I, Messier C, Kembel SW. Host species identity, site and time drive temperate tree phyllosphere bacterial community structure. Microbiome. 2016;4(1):27. 10.1186/s40168-016-0174-1. PMC4912770 27316353

[42] Li M, Hong L, Ye W, Wang Z, Shen H. Phyllosphere bacterial and fungal communities vary with host species identity, plant traits and seasonality in a subtropical forest. Environ Microbiome. 2022;17(1):29. 10.1186/s40793-022-00423-3. PMC9185928 35681245

[43] Burghard V, Wende S, Schneck V, Ulrich A. Inoculation of Fraxinus excelsior seedlings with bacterial strains to enhance the tolerance against ash dieback. J Plant Dis Prot. 2024;132(1):9. 10.1007/s41348-024-01014-9.

[44] Wagner MR, Lundberg DS, del Rio TG, Tringe SG, Dangl JL, Mitchell-Olds T. Host genotype and age shape the leaf and root microbiomes of a wild perennial plant. Nat Commun. 2016;7(1):12151. 10.1038/ncomms12151. PMC4945892 27402057

[45] Wang L, Liu Z, Bres C, Jin G, Fanin N. Coniferous Tree Species Identity and Leaf Aging Alter the Composition of Phyllosphere Communities Through Changes in Leaf Traits. Microb Ecol. 2024;87(1):126. 10.1007/s00248-024-02440-w. PMC11464569 39382725

[46] Pang Z, Chen J, Wang T, Gao C, Li Z, Guo L, Xu J, Cheng Y. Linking plant secondary metabolites and plant microbiomes: a review. [Review]. Front Plant Sci. 2021;12. 10.3389/fpls.2021.621276. PMC7961088 33737943

[47] Guerfel M, Baccouri O, Boujnah D, Chaïbi W, Zarrouk M. Impacts of water stress on gas exchange, water relations, chlorophyll content and leaf structure in the two main Tunisian olive (Olea europaea L.) cultivars. Sci Hort. 2009;119(3):257–63. 10.1016/j.scienta.2008.08.006.

[48] Francioli D, Cid G, Hajirezaei M-R, Kolb S. Response of the wheat mycobiota to flooding revealed substantial shifts towards plant pathogens. Front Plant Sci. 2022b;13–2022. 10.3389/fpls.2022.1028153. PMC9742542 36518495

[49] Al Ashhab A, Meshner S, Alexander-Shani R, Dimerets H, Brandwein M, Bar-Lavan Y, Winters G. Temporal and spatial changes in phyllosphere microbiome of acacia trees growing in arid environments. Front Microbiol. 2021;12. 10.3389/fmicb.2021.656269. PMC8312645 34322096

[50] Finkel OM, Burch AY, Elad T, Huse SM, Lindow SE, Post AF, Belkin S. Distance-Decay Relationships Partially Determine Diversity Patterns of Phyllosphere Bacteria on Tamrix Trees across the Sonoran Desert. Appl Environ Microbiol. 2012;78(17):6187–93. 10.1128/AEM.00888-12. PMC3416633 22752165

[51] Stone BWG, Jackson CR. Biogeographic Patterns Between Bacterial Phyllosphere Communities of the Southern Magnolia (Magnolia grandiflora) in a Small Forest. Microb Ecol. 2016;71(4):954–61. 10.1007/s00248-016-0738-4. 26883131

[52] Jia T, Yao Y, Guo T, Wang R, Chai B. Effects of plant and soil characteristics on phyllosphere and rhizosphere fungal communities during plant development in a copper tailings Dam. Front Microbiol. 2020;11. 10.3389/fmicb.2020.556002. PMC7550642 33133030

