# Supplementary material for: The core taxa connectivity in leaf microbiomes in ash trees stands is modulated by host-related and environmental factors
Source: Environ Microbiome. 2026 Oct 7;21:119. doi: 10.1186/s40793-026-00967-8 (PMC13651322; doi:10.1186/s40793-026-00967-8)
Supplement: Supplementary file 1 [file 40793_2026_967_MOESM1_ESM.docx]

Supplement Fig. 1 Relative proportions of the 40 largest bacterial genera in the microbiomes of ash trees, divided according to the sampling site. Information about the sampling sites is given in Table 1.

Supplement Fig. 2: Principal Coordinates Analysis (PCoA) plot depicting the ordination of microbiome samples on the basis of Bray‒Curtis dissimilarity. The analysis was performed on log-transformed ASV counts. The samples are colored according to their site, with each point representing a distinct microbiome sample.

Supplement Table 1: Alpha diversity indices of the microbiome composition obtained for the different sites (mean ± standard deviation).

| Site | Richness | Chao1 | Shannon | Simpson |
| --- | --- | --- | --- | --- |
| BB1 | 301±89.3 | 333±105 | 4.03±0.28 | 0.961±0.010 |
| BW1 | 284±90.4 | 312±108 | 3.94±0.33 | 0.957±0.018 |
| BY1 | 311±121 | 348±142 | 3.84±0.38 | 0.947±0.021 |
| BY2 | 369±135 | 408±159 | 4.05±0.43 | 0.957±0.019 |
| BY3 | 485±142 | 540±166 | 4.23±0.27 | 0.957±0.009 |
| HE1 | 357±105 | 383±120 | 4.12±0.44 | 0.958±0.018 |
| MV1 | 408±131 | 434±147 | 4.29±0.52 | 0.954±0.033 |
| NI1 | 287±70.1 | 320±80.0 | 3.81±0.34 | 0.950±0.020 |
| SN1 | 262±124 | 288±138 | 3.93±0.44 | 0.956±0.036 |
| ST1 | 231±64.4 | 245±73.2 | 3.78±0.31 | 0.951±0.020 |
| TH1 | 254±55.1 | 274±68.5 | 3.78±0.31 | 0.948±0.019 |
| TH2 | 283±120 | 307±135 | 4.00±0.29 | 0.963±0.009 |

Supplement Table 2: Network properties of the molecular ecological network at the ASV level according to the RMT-based approach.

| Network indexes | BB1 | BW1 | BY1 | BY2 | BY3 | HE1 | MV1 | NI1 | SN1 | ST1 | TH1 | TH2 |
| --- | --- | --- | --- | --- | --- | --- | --- | --- | --- | --- | --- | --- |
| Total nodes | 109 | 125 | 117 | 170 | 294 | 117 | 99 | 108 | 90 | 59 | 75 | 92 |
| Total links - Number of edges | 137 | 251 | 168 | 524 | 1342 | 146 | 117 | 213 | 100 | 58 | 90 | 115 |
| Edge-node ratio | 1.26 | 2.01 | 1.44 | 3.08 | 4.56 | 1.25 | 1.18 | 1.97 | 1.11 | 0.98 | 1.20 | 1.25 |
| Maximal degree | 9 | 27 | 15 | 36 | 42 | 10 | 7 | 23 | 9 | 7 | 7 | 14 |
| Average degree (avgK) | 2.51 | 4.02 | 2.87 | 6.17 | 9.13 | 2.50 | 2.36 | 3.94 | 2.22 | 1.97 | 2.4 | 2.5 |
| Network density (D) | 0.023 | 0.032 | 0.025 | 0.036 | 0.031 | 0.022 | 0.024 | 0.037 | 0.025 | 0.034 | 0.032 | 0.027 |
| R square of power-law | 0.83 | 0.839 | 0.942 | 0.83 | 0.726 | 0.944 | 0.867 | 0.736 | 0.881 | 0.922 | 0.84 | 0.918 |
| Network hubs | - | 1 | - | 1 | - | - | - | 1 | - | - | - | - |
| Modular hubs | 2 | 1 | 3 | 5 | 8 | 3 | - | 1 | 2 | - | - | 3 |
| Connectors | 4 | 14 | 3 | 18 | 40 | 7 | 2 | 6 | - | - | - | 1 |

Supplement Table 3: All used R packages and their citation

| R package | Abb. citation | Reference-list entry |
| --- | --- | --- |
| phangorn | Schliep (2011) | Schliep, K. P. (2011). phangorn: Phylogenetic analysis in R. *Bioinformatics, 27*(4), 592–593. doi:10.1093/bioinformatics/btq706. ([OUP Academic](https://academic.oup.com/bioinformatics/article/27/4/592/198887?utm_source=chatgpt.com)) |
| DECIPHER | Wright (2016) | Wright, E. S. (2016). Using DECIPHER v2.0 to analyze big biological sequence data in R. *The R Journal, 8*(1), 352–359. doi:10.32614/RJ-2016-025. ([The R Journal](https://journal.r-project.org/articles/RJ-2016-025/?utm_source=chatgpt.com)) |
| ggplot2 | Wickham (2016) | Wickham, H. (2016). *ggplot2: Elegant Graphics for Data Analysis*. Springer-Verlag New York. ISBN 978-3-319-24277-4. ([ggplot2](https://ggplot2.tidyverse.org/authors.html?utm_source=chatgpt.com)) |
| phyloseq | McMurdie and Holmes (2013) | McMurdie, P. J., & Holmes, S. (2013). phyloseq: An R package for reproducible interactive analysis and graphics of microbiome census data. *PLOS ONE, 8*(4), e61217. doi:10.1371/journal.pone.0061217. ([PubMed](https://pubmed.ncbi.nlm.nih.gov/23630581/?utm_source=chatgpt.com)) |
| vegan | Oksanen et al. (2026) | Oksanen, J., Simpson, G. L., Blanchet, F. G., Kindt, R., Legendre, P., Minchin, P. R., O’Hara, R. B., Solymos, P., Stevens, M. H. H., Szoecs, E., Wagner, H., et al. (2026). *vegan: Community Ecology Package*. R package version 2.8-0. doi:10.32614/CRAN.package.vegan. ([Vegan Devs](https://vegandevs.github.io/vegan/authors.html?utm_source=chatgpt.com)) |
| tidyverse | Wickham et al. (2019) | Wickham, H., Averick, M., Bryan, J., Chang, W., McGowan, L. D., François, R., Grolemund, G., Hayes, A., Henry, L., Hester, J., et al. (2019). Welcome to the tidyverse. *Journal of Open Source Software, 4*(43), 1686. doi:10.21105/joss.01686. ([CRAN](https://cran.r-project.org/web/packages/tidyverse/citation.html)) |
| microbiome | Lahti and Shetty (2017) | Lahti, L., & Shetty, S. (2017). *microbiome R package*. Bioconductor. doi:10.18129/B9.bioc.microbiome. ([Groningen Forschungsportal](https://research.rug.nl/en/publications/microbiome-r-package/?utm_source=chatgpt.com)) |
| microViz | Barnett et al. (2021) | Barnett, D. J. M., Arts, I. C. W., & Penders, J. (2021). microViz: An R package for microbiome data visualization and statistics. *Journal of Open Source Software, 6*(63), 3201. doi:10.21105/joss.03201. ([david-barnett.github.io](https://david-barnett.github.io/microViz/authors.html)) |
| multcompView | Graves et al. (2026) | Graves, S., Piepho, H.-P., & Selzer, L. (2026). *multcompView: Visualizations of Paired Comparisons*. R package version 0.1-12. ([R-universe](https://lselzer.r-universe.dev/multcompView?utm_source=chatgpt.com)) |
| rcompanion | Mangiafico (2026) | Mangiafico, S. S. (2026). *rcompanion: Functions to Support Extension Education Program Evaluation*. Rutgers Cooperative Extension, New Brunswick, New Jersey. Version 2.5.2. ([CRAN](https://cran.r-project.org/web/packages/rcompanion/citation.html)) |
| FSA | Ogle et al. (2026) | Ogle, D. H., Doll, J. C., Wheeler, A. P., & Dinno, A. (2026). *FSA: Simple Fisheries Stock Assessment Methods*. R package version 0.10.1. doi:10.32614/CRAN.package.FSA. ([CRAN](https://cran.r-project.org/web/packages/FSA/citation.html)) |
| pairwiseAdonis | Martinez Arbizu (2020) | Martinez Arbizu, P. (2020). *pairwiseAdonis: Pairwise multilevel comparison using adonis*. R software package, version 0.4. ([GitHub](https://github.com/pmartinezarbizu/pairwiseadonis?utm_source=chatgpt.com)) |
| ape | Paradis and Schliep (2019) | Paradis, E., & Schliep, K. (2019). ape 5.0: An environment for modern phylogenetics and evolutionary analyses in R. *Bioinformatics, 35*, 526–528. doi:10.1093/bioinformatics/bty633. ([CRAN](https://cran.r-project.org/web/packages/ape/citation.html)) |
| corncob | Martin et al. (2020) | Martin, B. D., Witten, D., & Willis, A. D. (2020). Modeling microbial abundances and dysbiosis with beta-binomial regression. *The Annals of Applied Statistics, 14*(1), 94–115. doi:10.1214/19-AOAS1283. ([PubMed Central (PMC)](https://pmc.ncbi.nlm.nih.gov/articles/PMC7514055/?utm_source=chatgpt.com)) |
| correlation | Makowski et al. (2020) | Makowski, D., Ben-Shachar, M. S., Patil, I., & Lüdecke, D. (2020). Methods and algorithms for correlation analysis in R. *Journal of Open Source Software, 5*(51), 2306. doi:10.21105/joss.02306. ([CRAN](https://cran.r-project.org/web/packages/correlation/citation.html)) |
| corrplot | Wei and Simko (2024) | Wei, T., & Simko, V. (2024). *R package “corrplot”: Visualization of a Correlation Matrix*. Version 0.95. ([GWDG](https://ftp.gwdg.de/pub/misc/cran/web/packages/corrplot/citation.html?utm_source=chatgpt.com)) |
| fastANCOM | Zhou et al. (2022) | Zhou, C., Wang, H., Zhao, H., & Wang, T. (2022). fastANCOM: A fast method for analysis of compositions of microbiomes. *Bioinformatics, 38*(7), 2039–2041. doi:10.1093/bioinformatics/btac060. ([PubMed](https://pubmed.ncbi.nlm.nih.gov/35134120/?utm_source=chatgpt.com)) |
| GUniFrac | Chen et al. (2012) | Chen, J., Bittinger, K., Charlson, E. S., Hoffmann, C., Lewis, J., Wu, G. D., Collman, R. G., Bushman, F. D., & Li, H. (2012). Associating microbiome composition with environmental covariates using generalized UniFrac distances. *Bioinformatics, 28*(16), 2106–2113. doi:10.1093/bioinformatics/bts342. ([PubMed](https://pubmed.ncbi.nlm.nih.gov/22711789/?utm_source=chatgpt.com)) |
| dplyr | Wickham et al. (2026) | Wickham, H., François, R., Henry, L., Müller, K., & Vaughan, D. (2026). *dplyr: A Grammar of Data Manipulation*. R package version 1.2.1. ([Dplyr](https://dplyr.tidyverse.org/authors.html?utm_source=chatgpt.com)) |
| microeco | Liu et al. (2026) | Liu, C., Li, X., Mansoldo, F. R. P., Chen, T., Meng, F., Tang, R., Zhou, S., Yang, Q., Shao, R., & Yao, M. (2026). microeco 2: A comprehensive R package for downstream analysis of microbiome omics data. *iMeta, 5*, e70132. doi:10.1002/imt2.70132. ([CRAN](https://cran.r-project.org/web/packages/microeco/citation.html?utm_source=chatgpt.com)) |
| file2meco | Liu et al. (2022) | Liu, C., Li, X., Mansoldo, F. R. P., An, J., Kou, Y., Zhang, X., Wang, J., Zeng, J., Vermelho, A. B., & Yao, M. (2022). Microbial habitat specificity largely affects microbial co-occurrence patterns and functional profiles in wetland soils. *Geoderma, 418*, 115866. doi:10.1016/j.geoderma.2022.115866. ([Illinois Experts](https://experts.illinois.edu/en/publications/microbial-habitat-specificity-largely-affects-microbial-co-occurr?utm_source=chatgpt.com)) |
| ecodist | Goslee and Urban (2007) | Goslee, S. C., & Urban, D. L. (2007). The ecodist package for dissimilarity-based analysis of ecological data. *Journal of Statistical Software, 22*(7), 1–19. doi:10.18637/jss.v022.i07. ([CRAN](https://cran.r-project.org/web/packages/ecodist/citation.html?utm_source=chatgpt.com)) |
| lme4 | Bates et al. (2015) | Bates, D., Mächler, M., Bolker, B. M., & Walker, S. C. (2015). Fitting linear mixed-effects models using lme4. *Journal of Statistical Software, 67*(1), 1–48. doi:10.18637/jss.v067.i01. ([CRAN](https://cran.r-project.org/web/packages/lme4/citation.html?utm_source=chatgpt.com)) |
| ggClusterNet | Wen et al. (2022) | Wen, T., Xie, P., Yang, S., Niu, G., Liu, X., Ding, Z., Xue, C., Liu, Y.-X., Shen, Q., & Yuan, J. (2022). ggClusterNet: An R package for microbiome network analysis and modularity-based multiple network layouts. *iMeta, 1*, e32. doi:10.1002/imt2.32. ([GitHub](https://github.com/taowenmicro/ggClusterNet?utm_source=chatgpt.com)) |
| NetCoMi | Peschel et al. (2021) | Peschel, S., Müller, C. L., von Mutius, E., Boulesteix, A.-L., & Depner, M. (2021). NetCoMi: Network construction and comparison for microbiome data in R. *Briefings in Bioinformatics, 22*(4), bbaa290. doi:10.1093/bib/bbaa290. ([PubMed](https://pubmed.ncbi.nlm.nih.gov/33264391/?utm_source=chatgpt.com)) |
| randomForest | Liaw and Wiener (2002) | Liaw, A., & Wiener, M. (2002). Classification and regression by randomForest. *R News, 2*(3), 18–22. ([cran.usk.ac.id](https://cran.usk.ac.id/web/packages/randomForest/citation.html?utm_source=chatgpt.com)) |
| caret | Kuhn (2008) | Kuhn, M. (2008). Building predictive models in R using the caret package. *Journal of Statistical Software, 28*(5), 1–26. doi:10.18637/jss.v028.i05. ([CRAN](https://cran.ms.unimelb.edu.au/web/packages/caret/citation.html?utm_source=chatgpt.com)) |
| readxl | Wickham and Bryan (2026) | Wickham, H., & Bryan, J. (2026). *readxl: Read Excel Files*. R package version 1.5.0. ([readxl.tidyverse.org](https://readxl.tidyverse.org/authors.html?utm_source=chatgpt.com)) |
| fastDummies | Kaplan (2026) | Kaplan, J. (2026). *fastDummies: Fast Creation of Dummy (Binary) Columns and Rows from Categorical Variables*. R package version 1.7.6. doi:10.32614/CRAN.package.fastDummies. ([R-universe](https://jacobkap.r-universe.dev/fastDummies?utm_source=chatgpt.com)) |
| reshape2 | Wickham (2007) | Wickham, H. (2007). Reshaping data with the reshape package. *Journal of Statistical Software, 21*(12), 1–20. doi:10.18637/jss.v021.i12. ([Journal of Statistical Software](https://www.jstatsoft.org/v21/i12/?utm_source=chatgpt.com)) |
